# The interplay of central insulin and menstrual cycle on functional brain networks and neural food cue reactivity in women

**DOI:** 10.1038/s42003-025-09341-9

**Published:** 2026-01-06

**Authors:** Julia Hummel, Sixiu Zhao, Sarah Sobotta, Ralf Veit, Leontine Sandforth, Andreas L. Birkenfeld, Andreas Fritsche, Robert Wagner, Andreas Peter, Hubert Preissl, Martin Heni, Stephanie Kullmann

**Affiliations:** 1 Institute for Diabetes Research and Metabolic Diseases of the Helmholtz Center Munich at the University of Tübingen, Tübingen, Germany; 2 Division of Endocrinology and Diabetology, Department of Internal Medicine I, University of Ulm, Ulm, Germany; 3 German Center for Diabetes Research (DZD), Neuherberg, Germany; 4 Department of Internal Medicine IV, Diabetology, Endocrinology and Nephrology, Eberhard Karls University Tübingen, Tübingen, Germany; 5 Department of Endocrinology and Diabetology, Medical Faculty and University Hospital Düsseldorf, Düsseldorf, Germany; 6 Institute for Clinical Diabetology, German Diabetes Center, Leibniz Center for Diabetes Research at Heinrich Heine University, Düsseldorf, Germany; 7 Institute for Clinical Chemistry and Pathobiochemistry, Department for Diagnostic Laboratory Medicine, Eberhard Karls University Tübingen, Tübingen, Germany

**Keywords:** Obesity, Feeding behaviour

## Abstract

The menstrual cycle impacts food intake, peripheral metabolism, and brain function. One well-known central regulator of eating behavior is the hormone insulin. Here, we show that the responsiveness of functional brain networks to central insulin varies dynamically across the menstrual cycle in premenopausal women. Intranasal insulin (INI) administration increases functional connectivity within networks that support decision-making processes (namely the default mode and salience network) in the follicular compared to the luteal phase of the menstrual cycle. In contrast, INI decreases functional connectivity within the somatosensory network during the follicular phase relative to the luteal phase. In response to visual food cues, hippocampus and dorsal striatum activity are higher in the luteal compared to the follicular phase, particularly to sweet food. Estradiol and progesterone levels predict these changes. This could contribute to higher food craving and food intake observed in the luteal phase. Our findings emphasize sex hormones’ role in modulating brain sensitivity to hormonal signals and external stimuli.

## Abstract (web summary)

Central insulin effects on brain networks and food cue reactivity in the hippocampus and striatum vary dynamically across the menstrual cycle in premenopausal women, with estradiol and progesterone predicting these changes.

## Introduction

Eating behavior changes across the menstrual cycle in females, with higher food intake in the luteal phase compared to follicular phase^1^. This phenomenon is often attributed to hormonal fluctuations during the menstrual cycle, with appetite-reducing effects of estrogen and/or appetite-enhancing effects of progesterone^2^. Furthermore, peripheral insulin sensitivity adapts during the menstrual cycle with relative peripheral insulin resistance in the luteal phase^3^. Recent evidence indicates that changes in brain insulin responsiveness during the luteal phase may contribute to this effect^4,5^.

Central insulin-mediated effects regulate food intake and peripheral metabolism in an anorexigenic fashion^6^. Thereby, brain insulin responsiveness can determine body weight and body composition over a course of 10 years^7^. Furthermore, the disruption of insulin responsiveness in the human brain promotes, besides metabolic, also psychiatric and neurodegenerative diseases^6^. Intranasal insulin (INI) is a non-invasive method for rapidly delivering insulin to the brain via nasal spray while minimizing systemic absorption^8,9^. Neuroimaging studies using INI administration to study central insulin-mediated effects suggest that insulin modulates reward processing via the mesolimbic circuitry^6,10^. Specifically, INI inhibits blood oxygen level-dependent (BOLD) responses in mesolimbic regions, both during resting-state and food cue exposure^8,11–16^. These neural changes were associated with reduced food craving and sweet foods preference^8,17^. Additionally, INI reduced hunger by enhancing functional connectivity in the hippocampus, further supporting its anorexigenic effects^18^. Individuals with obesity-associated peripheral insulin resistance, on the other hand, showed disrupted central insulin action, along with a higher preference for palatable foods^13,17,19,20^.

Notably, central insulin’s anorexigenic effect appears to be sex-specific, with acute INI administration reducing postprandial snack intake in females and fasting intake in males, while chronic administration reduced body weight only in males^21–23^. Moreover, females exhibited a stronger insulin-induced neural food cue response in the dorsolateral prefrontal cortex (dlPFC)^14^. Given these sex differences, sex hormones and their fluctuation across the female menstrual cycle might play an important role^24,25^. For example, prior work in animal models suggests that estrogen may contribute more prominently to sex-specific differences in central insulin sensitivity^26^. In addition, brain responses to food cues, particularly high-caloric food, vary across the menstrual cycle in reward processing regions - mesolimbic circuitry, including the striatum, hippocampus, amygdala, and insula^27–30^. Additionally, sex hormones modulate functional connectivity (FC) of brain networks, particularly in the default mode network (DMN), salience network (SN), and executive control network (ECN) throughout the menstrual cycle^31–36^.

Nevertheless, it remains unclear how the menstrual cycle modulates central insulin action within large-scale functional brain networks and influences the brain activity’s response to visual food cues.

**Fig. 1:**
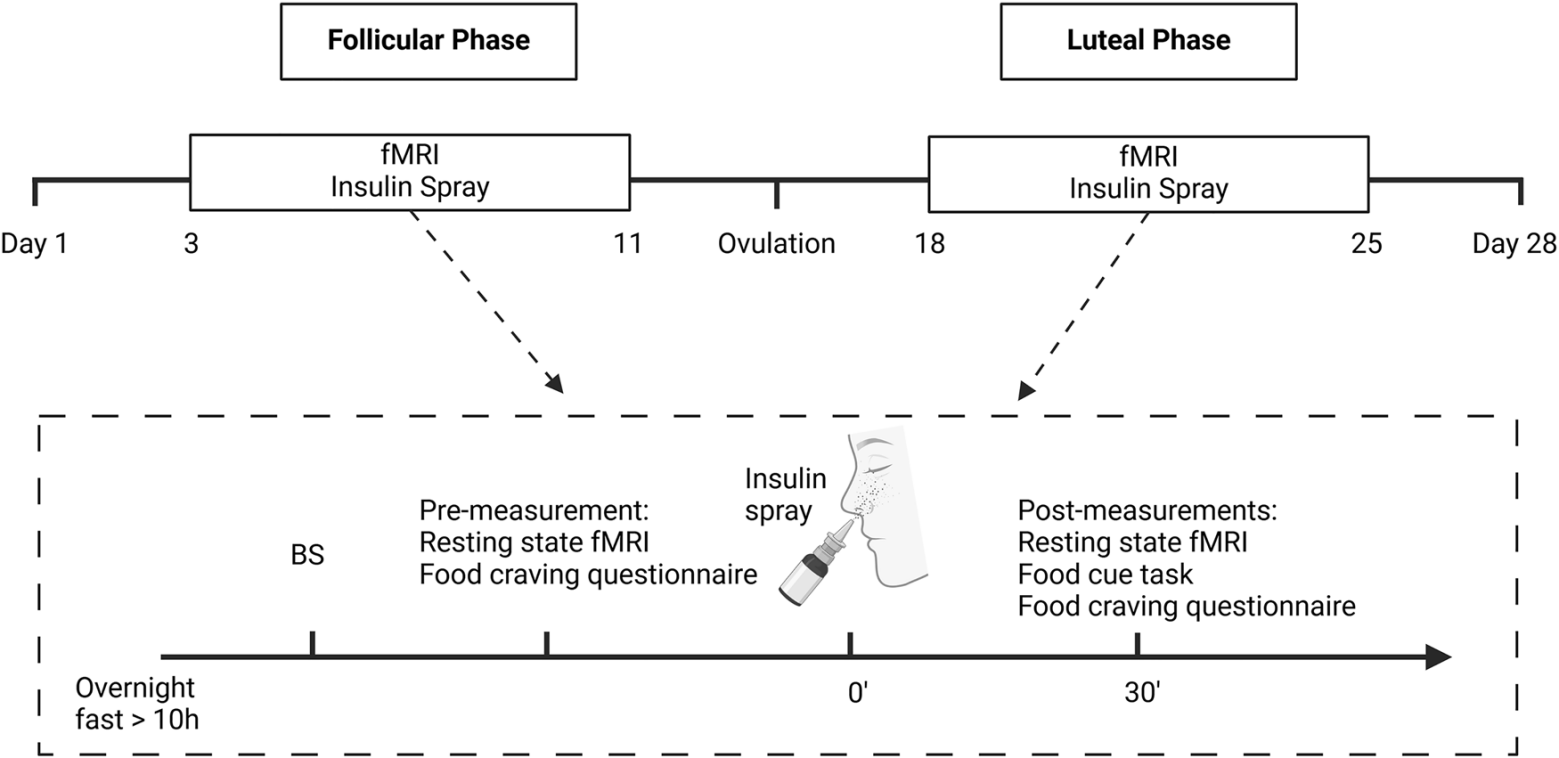
Study design. After an overnight fast, participants underwent two fMRI sessions in a randomized design—once during the follicular and once during the luteal phase of the menstrual cycle. Resting-state fMRI and food craving assessments were conducted at baseline and after intranasal insulin administration, followed by a visual food cue task performed in the scanner after nasal insulin. BS blood sample, fMRI functional magnetic resonance imaging. Adapted from ref. ^4^.

In this study, we examined the interaction of menstrual phases and central insulin on functional brain networks and neural food cue reactivity. Using a within-subject design, fifteen females with natural menstrual cycles underwent functional magnetic resonance imaging (fMRI) measurements in the follicular and luteal phases of the menstrual cycle, before and after INI administration (for study design, see Fig. 1). FC of brain networks was assessed using resting-state fMRI with group independent component analysis (ICA). The BOLD signal was measured in response to visual food cues to evaluate neural food cue reactivity. We hypothesized increased FC in response to INI in functional brain networks related to cognition and reward processes and lower neural food cue reactivity in insulin-sensitive brain areas during the follicular phase compared to the luteal phase^4,31–36^. Finally, as an exploratory analysis, we investigated whether estradiol, progesterone, or both hormone levels drive the cycle phase–specific effects on insulin-mediated resting-state network functional connectivity and food cue reactivity. Clarifying how central insulin action varies across the menstrual cycle may improve our understanding of sex-specific neuroendocrine mechanisms underlying metabolic regulation and eating behavior.

## Results

### Participants

Women examined in this study had a regular menstrual cycle of 29 days [IQR: 27, 30] and a median age of 23 years [IQR: 21, 26]. Their median BMI was 21.8 kg/m² [IQR: 20.5, 22.7] and they had normal glucose control (HbA1c: 33 mmol/mol [IQR: 31, 35]).

There were no changes in BMI between the cycle phases (t^14^ = 0.70, 95% CI [−0.16, 0.32], mean diff = 0.08, d = 0.18, *p* = 0.5). Sex hormone concentrations differed between phases, with higher estradiol (t^14^ = −3.72, 95% CI [−1.03, −0.28], mean diff = −0.65, d = −0.96, *p* = 0.002) and progesterone levels (t^14^ = −7.96, 95% CI [−2.95, −1.70], mean diff = −2.32, d = −2.06, *p* < 0.0001) in the luteal phase. The ratio between estradiol and progesterone (E/P ratio) was higher in the follicular phase (t^13^ = 5.17, 95% CI [0.91, 2.22], mean diff = 1.57, d = 1.38, *p* < 0.001, Table 1, Supplementary Fig. S1).

**Table 1:** Participant characteristics*

	Follicular Phase	Luteal Phase	*p*
	*N* = 15	*N* = 15	
**BMI (kg/m**^**2**^**)**	21.8 (1.72)	21.7 (1.45)	0.5
**Estradiol (pmol/L)**	264.0 (173.0)	566.0 (361.0)	**0.002**
**Progesterone (nmol/L)**	2.0 (0.70)	28.5 (25.2)	**<0.001**
**Estradiol/ Progesterone ratio**	110.0 (54.6)	19.9 (17.2)	**<0.001**

*BMI* body mass index. * Data are presented as median (interquartile range).

### Resting-state fMRI

#### Effect of cycle phase and intranasal insulin on resting-state functional connectivity

fMRI measurements were conducted in a randomized, cross-over design on two separate days: one during the follicular phase and one during the luteal phase of the menstrual cycle (Fig. 1). Resting-state fMRI scans were conducted at baseline and 30 min after INI on each fMRI measurement day. Twenty resting-state functional networks were investigated for changes based on menstrual cycle and insulin administration (Supplementary Fig. S2).

Differences in FC between cycle phases were observed within the posterior DMN, cerebellar network and salience network. Specifically, higher FC in the left orbitofrontal cortex within the posterior DMN (*p*_FWE_ (small volume correction (SVC)) = 0.03, Table 2) and in the left putamen within the cerebellar network (*p*_FWE_ (SVC) = 0.039, Table 2) was found in the follicular phase compared to luteal phase. Conversely, the right insula within the salience network exhibited higher FC in the luteal phase relative to the follicular phase (*p*_FWE_ (SVC) = 0.048, Table 2). However, these results did not survive multiple comparison correction (*p* < 0.01 corrected for the number of ROIs for SVC).

**Table 2:** Effect of cycle phase and intranasal insulin on resting-state functional connectivity

			MNI coordinates					
Network	Brain region	Hemi	x	y	z	Peak t	Cluster size	*p*_FWE_
**Effect of Cycle Phase: Follicular > Luteal**								
Posterior Default Mode Network								
	Medial OFC	L	−8	44	−10	4.23	90	*0.030^SVC^
Cerebellar Network								
	Putamen	L	−26	−10	6	4.56	54	*0.039^SVC^
**Effect of Cycle Phase: Luteal > Follicular**								
Salience Network								
	Insula	R	40	−10	0	4.41	21	*0.048^SVC^
**Effect of Spray: Post > Pre**								
Left Executive Control Network								
	Lingual gyrus	L	−10	−70	−4	5.06	120	0.027
**Interaction of cycle phase × pre vs. post insulin spray**								
Salience Network								
	Putamen	L	−20	−8	0	5.97	196	0.001
Somatosensory Network								
	SMA	R	4	2	52	4.89	138	0.014
Anterior Default Mode Network								
	Hippocampus	R	28	−30	−8	4.33	56	0.007^SVC^

*FWE* family-wise error; Hemi, hemisphere, *L* left, *R* right, *SMA* supplementary motor area. *p*-value FWE corrected using whole-brain cluster correction; SVC *p*_FWE_ small volume corrected for the ROIs. * Do not survive Bonferroni correction *p* < 0.01.

Differences in FC between pre versus post insulin administration were observed in the lingual gyrus within the left executive control network (*p*_FWEc_ = 0.027, whole-brain level corrected, Table 2).

Differences in FC based on insulin administration and cycle phase (i.e., interaction effect) were observed within the salience network (*p*_FWEc_ = 0.001, whole-brain level corrected, Table 2, Supplementary Fig. S3A), somatosensory network (*p*_FWEc_ = 0.014, whole-brain level corrected, Table 2, Supplementary Fig. S3B) and anterior DMN (*p*_FWE_ (SVC) = 0.007, Table 2, Supplementary Fig. S3C).

**Fig. 2:**
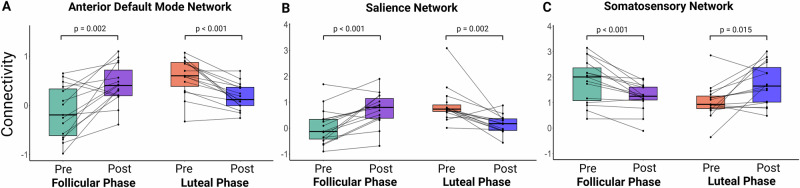
Interaction of menstrual cycle phase and intranasal insulin on resting-state functional connectivity. Box plots show that functional connectivity (FC) increased, from before (pre) to after (post) intranasal insulin administration, in the follicular phase and decreased in the luteal phase (**A**) in the right hippocampus of the anterior default mode network (interaction: *p*_FWE_ = 0.007, small volume correction) and **B** the left putamen of the salience network (interaction: *p*_FWE_ = 0.001). In contrast, FC decreased in the follicular phase and increased in the luteal phase from before to after intranasal insulin administration (**C**) in the supplementary motor area of the somatosensory network (interaction: *p*_FWE_ = 0.014). Presented are Tukey box plots with whiskers extending to 1.5 times the interquartile range and individual data points. *p* values in the figure are derived from two-sided paired t-tests (post-hoc) for comparison of functional connectivity measures before (pre) and after (post) spray administration for each phase. All analyses included *n* = 15 participants.

Post-hoc analysis revealed that following INI administration, FC increased in the right hippocampus of the anterior DMN (post-pre: t^14^_follicular_ = 3.91, *p*_follicular_ = 0.002; t^14^_luteal_ = −5.28, *p*_luteal_ < 0.001, Fig. 2A), and the right putamen of the salience network (post-pre: t^14^_follicular_ = 4.44, *p*_follicular_ < 0.001; t^14^_luteal_ = −3.95, *p*_luteal_ = 0.002, Fig. 2B) during the follicular phase but decreased during the luteal phase. In contrast, the supplementary motor area in the somatosensory network (post-pre: t^14^_follicular_ = −4.33, *p*_follicular_ < 0.001, Fig. 2C) showed a decrease in FC during the follicular phase and an increase during the luteal phase following INI administration.

#### Exploratory effects of sex hormones on resting-state functional connectivity networks

Fifteen participants were included in the estradiol and progesterone analyses, and 14 participants in the E/P ratio model due to exclusion of one outlier (>3 SD above the mean).

Independent of cycle phase and insulin administration, sex-hormones were significant predictors of resting-state functional connectivity. Specifically, progesterone and the E/P ratio predicted FC in the putamen within the cerebellar network (progesterone levels: estimate = −0.27, 95% CI [−0.38, −0.16], t^26^ = −5.22, *p* < 0.001) (E/P ratio: estimate = 0.38, 95% CI [0.23, 0.53], t^25^ = 5.11, *p* < 0.001). Moreover, circulating estradiol (estimate = 0.27, 95% CI [0.27, 1.15], t^26^ = 3.32, *p* = 0.003) as well as progesterone levels (estimate = 0.26, 95% CI [0.04, 0.47], t^26^ = 2.49, *p* = 0.020) predicted FC in the right insula within the salience network.

**Fig. 3:**
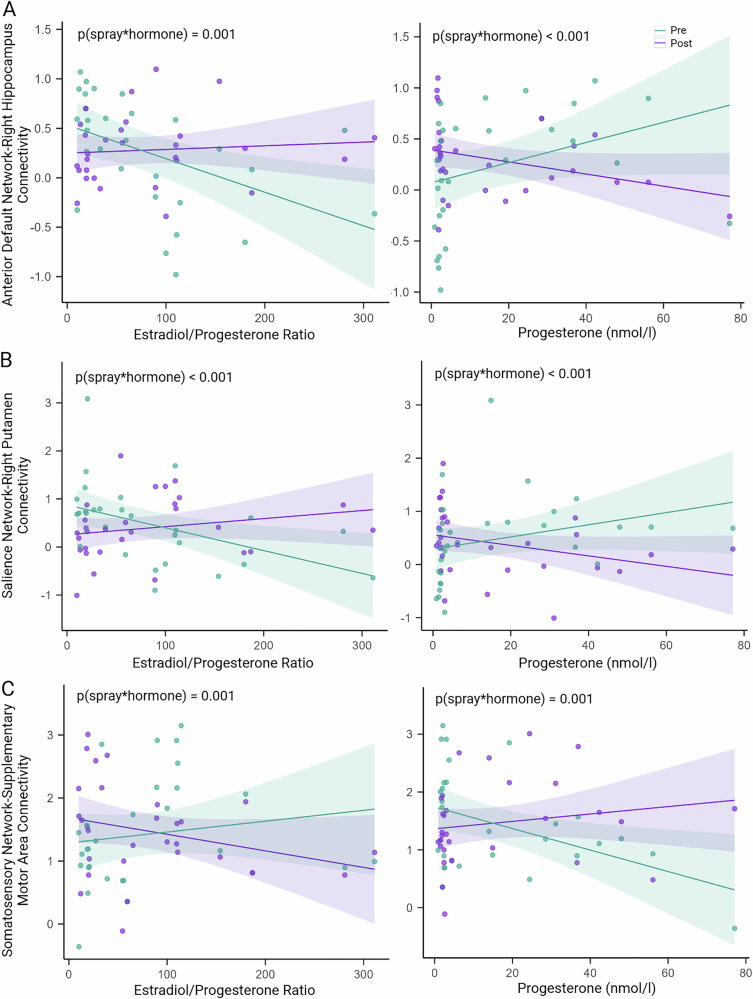
Interactions of sex hormone levels and insulin administration (pre vs. post) on resting-state functional connectivity. **A** In the right hippocampus of the anterior default mode network and **B** the left putamen of the salience network, a high estradiol/progesterone (E/P) ratio was linked to a higher functional connectivity (FC) after intranasal insulin administration, primarily driven by the level of progesterone. **C** In contrast, in the supplementary motor area of the somatosensory network, a high E/P ratio predicted lower FC after intranasal insulin, also driven by progesterone. *p-*values are derived from linear mixed models with log-transformed sex hormone levels. Analyses included *n* = 15 participants for progesterone models, and *n* = 14 for the E/P ratio model due to exclusion of one outlier (>3 SD above the mean).

We further examined possible interaction effects between sex hormone levels and the insulin spray effect (pre vs. post spray) on FC. An interaction was found for E/P ratio and progesterone and the INI effect on FC within the anterior DMN, the salience network, and the somatosensory network (all *p* ≤ 0.001, Table 3, Fig. 3A–C). In both the anterior DMN (Fig. 3A) and the salience network (Fig. 3B), models indicated that a high E/P ratio, as present during the follicular phase, was associated with increased insulin-induced FC. In turn, low E/P ratio, as present during the luteal phase, was associated with reduced insulin-induced functional connectivity. This negative correlation was primarily driven by progesterone levels. In the somatosensory network (Fig. 3C), on the contrary, this model suggests that a high E/P ratio predicted reduced FC after INI, an effect also driven by progesterone levels.

**Table 3:** Exploratory effects of sex hormones on resting-state functional connectivity networks

FC networks	E/P ratio*		Progesterone	
	Estimate (95% CI)	*p*_spray*hormone_	Estimate (95% CI)	*p*_spray*hormone_
Hippocampus - Anterior DMN	0.37 (0.16, 0.57)	0.001	−0.32 (−0.47, −0.16)	<0.001
Putamen - Salience Network	0.61 (0.32, 0.90)	< 0.001	−0.47 (−0.68, −0.26)	<0.001
SMA - somatosensory network	−0.52 (−0.82, −0.22)	0.001	0.40 (0.18, 0.61)	0.001

*CI* confidence interval, *DMN*, default mode network, *E* estradiol, *FC* functional connectivity, *P* progesterone; *SMA*, supplementary motor area. * One E/P ratio measurement was identified as outlier (>3 SD above mean) and was therefore excluded from analyses. Estradiol showed no interaction with intranasal insulin-induced FC (*p* > 0.1).

Estradiol showed no interaction with INI-induced FC in any of the networks (*p* > 0.1).

### Task-based fMRI results: Neural food cue reactivity

#### Whole-brain analysis

**Fig. 4:**
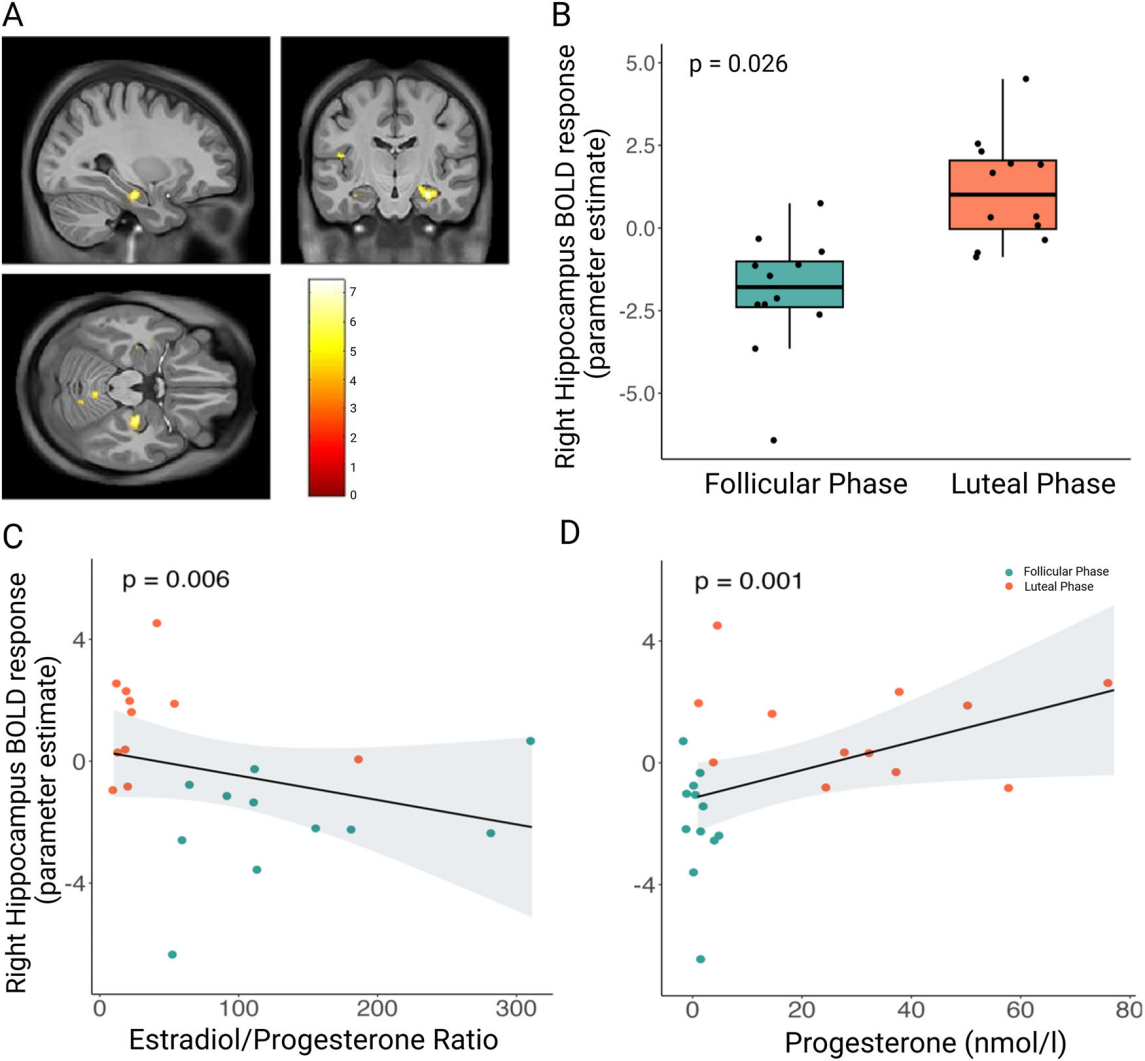
Effects of menstrual cycle phase on whole-brain activity during food cue task. **A** Activity in the right hippocampus in response to sweet vs. savory food cues was higher in the luteal phase compared to the follicular phase (*p*_FWE_ = 0.026, whole-brain corrected). Color map corresponds to t values (*p* < 0.001 uncorrected) overlaid on the standardized T1 image. **B** Box plot shows that the extracted BOLD response (beta values) of the right hippocampus was higher in the luteal phase compared to the follicular phase. Presented are tukey box plots with whiskers extending to 1.5 times the interquartile range and individual data points. **C** Correlation plot shows that a high estradiol/progesterone (E/P) ratio was linked to a lower hippocampal response after intranasal insulin administration. *p* values are derived from linear mixed models with log-transformed sex hormone levels. **D** Correlation plot shows that high progesterone levels were related to higher hippocampal response after intranasal insulin administration. Hence, the negative correlation between E/P ratio and hippocampal response was primarily driven by progesterone levels. Analyses included *n* = 12 participants for **A** and **B**, *n* = 11 for **C**, and *n* = 12 for **D**.

There were no significant differences between the follicular and luteal phases of the menstrual cycle in response to all food cues or to high-minus low-caloric food (*p*_FWEc_ > 0.05, whole-brain level). For the contrast sweet minus savory food images, the right hippocampus (peak-voxel (MNI) x: 28, y: −16, z: -20; *p*_FWEc_ = 0.026, whole-brain level corrected) showed higher response in the luteal phase compared to the follicular phase (Fig. 4A, B).

#### Exploratory ROI analysis of insulin-sensitive brain regions

**Fig. 5:**
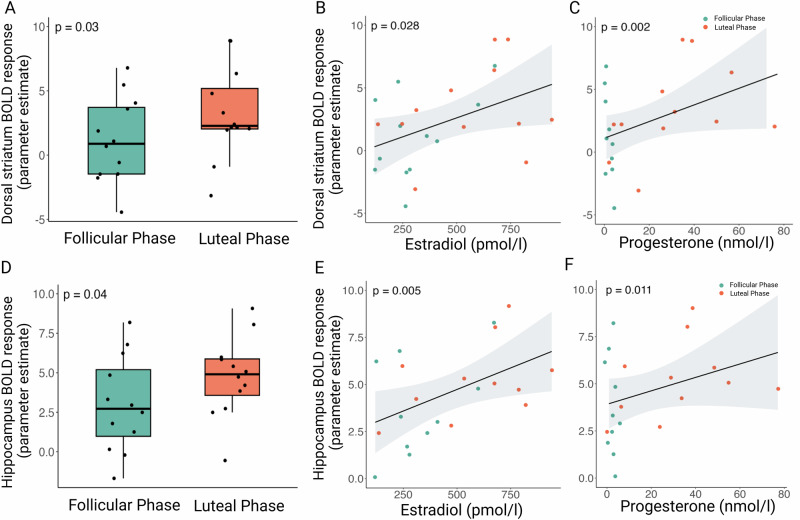
Effects of menstrual cycle phase on regions of interest during the food cue task. Box plot shows that the extracted BOLD responses to all food-cues in **A** bilateral dorsal striatum and **D** bilateral hippocampus were higher in the luteal phase compared to the follicular phase. Presented are Tukey box plots with whiskers extending to 1.5 times the interquartile range and individual data points. *p*-values are derived from two-sided paired t-tests for comparison of BOLD response between the phases. **B**, **C** Correlation plots show that high estradiol and progesterone levels positively correlated with activity in the dorsal striatum. **E**, **F** Similarly, high estradiol and progesterone levels were associated with increased hippocampal activity. *p*-values are derived from linear mixed models with log-transformed sex hormone levels. All analyses included *n* = 12 participants.

In response to all food cues, the bilateral dorsal striatum (t^11^ = −2.51, 95% CI [−3.91, −0.26], std. ß = 0.72, effect size = 0.60, mean diff. = 2.09, *p* = 0.029, Fig. 5A), and the bilateral hippocampus (t^11^ = −2.30, 95% CI [−3.39, −0.07], d = 0.62, std ß = 0.66, mean diff. = 1.73, *p* = 0.042, Fig. 5C) showed higher BOLD activity in the luteal compared to the follicular phase.

BOLD activity in response to high- minus low-caloric food cues showed no differences between the phases of the menstrual cycle (*p* > 0.05).

BOLD activity in response to sweet minus savory food cues showed a marginal difference in the bilateral hippocampus between the luteal and follicular phases. (t^11^ = −2.15, 95% CI [−1.65, 0.02], std. ß = 0.62, d = 0.67, mean diff. = 0.82, *p* = 0.054).

No differences were found between the phases in the amygdala, DLPFC, mOFC, insula, and ventral striatum (*p* > 0.05).

#### Exploratory findings of sex hormones effects on neural food cue reactivity

Twelve participants were included in the estradiol and progesterone analyses, and 11 participants in the E/P ratio model due to exclusion of one outlier (>3 SD above the mean).

In the right hippocampus, BOLD response to sweet food images was negatively linked to the E/P ratio (estimate = −1.12, 95% CI [−1.88, −0.36], t^11^ = −3.08, *p* = 0.006, (Fig. 4C), and positively to progesterone levels (estimate = 0.91, 95% CI [0.41, 1.40], t^12^ = 3.80, *p* = 0.001, Fig. 4D).

In the dorsal striatum, food cue reactivity to all food cues was positively associated with estradiol (estimate = 2.05, 95% CI [0.04, 0.70], t^16^ = 2.37, *p* = 0.028; Fig. 5B), progesterone (estimate = 0.96, 95% CI [0.38, 1.54], t^12^ = 3.48, *p* = 0.002; Fig. 5C), and the E/P ratio (estimate = −0.91, 95% CI [−1.77, −0.05], t^11^ = −2.21, *p* = 0.040).

Similarly, in the bilateral hippocampus, BOLD response to all food cues was positively correlated with estradiol (estimate = 2.15, 95% CI [0.16, 0.80], t^17^ = 3.14, *p* = 0.005; Fig. 5E) and progesterone levels (estimate = 0.74, 95% CI [0.19, 1.30], t^12^ = 2.79, *p* = 0.011; Fig. 5F).

#### Effect of cycle phase on food craving

Food desire was significantly stronger in the luteal phase compared to the follicular phase, independent of INI (t^42^ = 2.44, 95% CI [0.30, 2.90], mean diff. = 1.60, *p* = 0.019).

No significant differences were found between the phases for hunger ratings, the other FCQ-S subscales, or the total craving score, nor for the interaction between phase and INI (*p* > 0.05).

## Discussion

In the current study, we examined the effect of central insulin on resting-state functional brain networks and neural food cue reactivity across the menstrual cycle. Our findings suggest that the central insulin responsiveness of functional brain networks supporting decision-making processes varies dynamically across the menstrual cycle. As hypothesized, INI increased FC within the DMN and the SN during the follicular phase but decreased FC during the luteal phase. In contrast, FC within the somatosensory network decreased after INI administration in the follicular phase but increased in the luteal phase. Additionally, we observed a higher food cue reactivity in the hippocampus and dorsal striatum in the luteal phase compared to the follicular phase, particularly to sweet food. Estradiol and progesterone levels predicted FC in resting-state networks and food cue responses independent of cycle phase, further supporting the interaction between sex hormones and insulin action in the female brain.

Central insulin exhibited a cycle phase-dependent effect on major functional brain networks, including the DMN, SN, and somatosensory network. Specifically, networks related to cognition and reward responded with an increase after INI administration in the follicular but not luteal phase, while FC in the somatosensory network showed an opposite pattern. Independent of central insulin action, females showed a higher FC within the DMN and cerebellar network during the follicular phase, while the salience network exhibited higher FC in the luteal phase relative to the follicular phase. These findings suggest that brain functional connectivity and central insulin responsiveness of brain networks is changing across the menstrual cycle, with mainly reduced insulin responsiveness in the luteal phase. Concomitantly, we recently showed a reduced effect of central insulin action (i.e., hypothalamus) on peripheral insulin sensitivity in the luteal phase^4^, which could contribute to the relative peripheral insulin resistance previously reported^3^.

Indeed, peripheral insulin resistance has been suggested to be related to network FC, such that individuals with obesity-associated insulin resistance have weaker FC in regions of the DMN and SN, but stronger FC in the supplementary motor area (for reviews, see refs. ^6,37,38^). Furthermore, peripheral insulin resistance modulates insulin-induced FC in the nodes of the DMN and SN^13,20^. Moreover, the enhanced effect of INI on FC within the DMN, particularly in the hippocampus, has been reported before in both healthy young individuals^18^ as well as patients with type 2 diabetes mellitus^39^. Our findings replicated this effect in healthy premenopausal women, but only in the follicular phase, suggesting a menstrual phase-dependent effect of central insulin on functional brain networks in females. Taken together, it is plausible that relative brain insulin resistance extends beyond the hypothalamus during the luteal phase, affecting communication within large-scale brain networks.

Specifically, the DMN supports a range of internally focused mental processes, including self-reflection, memory, social understanding, and goal-directed behavior^38^. The SN, on the other hand, acts as the brain’s moderator, ensuring that attention and cognitive resources are allocated to the most important information, and coordinating the activity of other major brain networks to support adaptive behavior and decision-making^38,40^. In particular, within these networks, the hippocampus plays a role in both memory and interoceptive processes, such as hunger and satiety detection, whereas the putamen is critical within the SN in integrating sensory, cognitive, and motor information to guide behavior^41–43^. Altered FC within these networks has been speculated to reflect changes in response to satiety signals and influence food choices^38^. For example, FC of the DMN and SN negatively predicted carbohydrate consumption and eating disorders, such as disinhibited eating^44–46^. Concomitantly, females tend to experience greater carbohydrate cravings, more emotional eating, and higher energy intake during the luteal compared to the follicular phase^1,47,48^, which aligns with the higher food desire but not hunger found in the luteal phase in the current study. Thus, lower responsiveness to central insulin in the hippocampus of the DMN and putamen of the SN could potentially contribute to overeating behavior in females.

Central insulin has been shown to modulate mesolimbic activity among regions by an increased regional blood flow and functional connectivity in response to INI in regions involved in food reward processing and memory. For instance, INI increases blood flow in the striatum, including the putamen^49^ and reduces striatal dopaminergic signaling^11^. Hippocampus blood flow^50^ and functional connectivity^18,51^ is also increased by INI with a subsequent decrease in the desire to eat and food craving and lower palatability ratings of high-caloric foods^13,17^. Obesity associated insulin resistance, however, reduces the ability of central insulin to influence mesolimbic activity^13^. Hence, stronger insulin responsiveness in regions of mesolimbic circuitry, as observed in the follicular phase, could attenuate food reward and motivation to eat, highlighting the potential influence of menstrual phases on eating behavior.

Similarly, we observed a higher neural food cue response in the hippocampus and dorsal striatum, especially to sweet foods, during the luteal phase after nasal insulin. This is consistent with previous studies showing that brain responses to food cues vary across the menstrual cycle, especially in the mesolimbic circuitry^27,28^. For example, food cue reactivity in the dorsal striatum is higher in the luteal phase than the follicular phase in women with natural menstrual cycles, comparable to brain activity in women on progesterone-dominant contraceptives^27^. Additionally, sex differences in insulin-mediated food cue responses in the hippocampus have been observed, possibly reflecting hormonal influences on women’s hippocampus functional connectivity to food cues during the luteal phase^51^. Interestingly, similar to women in the luteal phase, individuals with obesity-related insulin resistance also exhibit increased activity in reward-related brain regions (for review, see ref. ^52^). Moreover, heightened neural food cue reactivity is linked to increased food cravings and even predicts weight gain^52–54^. These findings provide further insight into the neural mechanisms underlying different eating behaviors across the menstrual cycle.

We further observed the predictive role of sex hormones in insulin-induced brain network connectivity and neural response to food cues. This aligns with animal studies showing that sex hormones modulate central insulin sensitivity, influencing food intake and body weight^26^.

Sex hormone receptors are widely distributed throughout the brain, and animal studies have shown that their expression fluctuates across the estrous cycle^55,56^. In humans, these receptors are expressed in reward processing regions such as the mesolimbic dopamine pathway (for review, see ref. ^57^). In this circuit, dopamine is known to promote food-seeking behavior^58^, while central insulin reduces dopaminergic activity and suppresses food-related reward responses^11^. Meanwhile, sex hormones can modulate this system by influencing neurotransmission (e.g., dopamine) and receptor responsiveness, thereby shaping reward-related behaviors^57^. These mechanisms support the idea that fluctuations in sex hormone receptor expression across the menstrual cycle may modulate the interaction between sex hormone signaling and central insulin action, particularly in the mesolimbic pathway. Although the precise neurochemical mechanisms remain unclear, evidence suggests that estrogen and insulin/insulin-like growth factor-1 receptors may regulate each other’s expression and converge on shared intracellular cascades (e.g., MAPK, PI3K) in the brain^59,60^.

These interactions may help explain menstrual cycle–related changes in insulin-mediated brain connectivity and food-cue reactivity, as well as variations in eating behavior observed in previous studies^1,47,48^. However, further studies are needed to test these hypotheses and elucidate the mechanistic underpinnings in humans.

Interestingly, the inverse association pattern between insulin-induced brain alterations and both the E/P ratio and progesterone may indicate a more specific role of progesterone in central insulin action. Consistent with this, behavioral studies in humans found no effect of estradiol on either food intake or cognition after INI administration, again pointing towards stronger effects of progesterone rather than estradiol^61–63^. Our recent data, showing an association between food cue-induced hippocampal FC after INI and E/P ratio in the luteal phase only, further supports this idea^51^. However, the mechanisms of sex hormonal effects, especially progesterone, on central insulin action require further investigation. Comparisons between young women using progesterone-dominant contraceptives and postmenopausal women could help clarify progesterone’s modulatory role.

We present novel findings on the interplay between INI and the menstrual cycle in functional resting-state networks and neural food cue reactivity. However, some limitations of the current study should be acknowledged. First, no placebo nasal spray condition was used; hence, the food cue response was only evaluated under INI application. Given that expectancy effects of interventions can influence both subjective experiences (e.g., hunger, craving) and neural responses to food cues^64,65^, it is difficult to disentangle the specific effects of INI from participants’ expectations. A double-blind, placebo-controlled crossover design is warranted in future studies. Second, replication of the current results is necessary due to the small sample size. Third, whether the menstrual cycle phase influences neural food cue responses in the fasted versus satiated state remains to be investigated. Finally, the association between food intake, ingestive biomarkers (e.g., ghrelin), and central insulin-induced brain alterations across the menstrual cycle warrants further investigation.

In conclusion, our data reveal that the menstrual cycle significantly modulates the brain’s response to central insulin and visual food cues, with notable differences between the follicular and luteal phases. Insulin enhanced FC of functional brain networks supporting memory, attention, and decision-making processes only during the follicular phase, while neural food cue reactivity was stronger in the luteal phase, potentially driving increased food craving and food intake. Our findings underscore the important role of sex hormones in modulating the brain’s sensitivity to both internal hormonal signals and external stimuli.

## Methods

### Participants

Nineteen healthy premenopausal women with a natural menstrual cycle were recruited for the study. We included women without underlying diseases, who neither used contraceptives, nor any other medication (see ref. ^4^ for detailed inclusion and exclusion criteria). Four participants were excluded as they did not meet the inclusion criteria, declined participation, withdrew consent, or were not in the allocated cycle phase^4^. Therefore, 15 participants were included in the final analysis. For the task-based fMRI analysis, three participants were further excluded due to incomplete fMRI measurements or excessive head motion.

All ethical regulations relevant to human research participants were followed. The study protocol was approved by the Ethics Committee of the Medical Faculty at Eberhard Karls University of Tübingen and was pre-registered on ClinicalTrials.gov (https://clinicaltrials.gov/study/NCT03929419). Participants provided informed written consent before any study-related assessments. After providing written informed consent, participants underwent eligibility screening, which included a review of their medical history and collection of a blood sample.

The sample size was chosen based on previous within-subject crossover studies from our group using INI^66^, which reported moderate-to-high effects (Cohen’s *dz* = 0.7). The primary endpoint analysis showing menstrual cycle-specific effects on peripheral glucose metabolism has already been published (see ref. ^4^), we here report secondary outcomes.

### Study design

Participants were instructed to consume their last meal by 7:00 p.m. the evening before each visit and to drink only water afterward. They arrived at the study center in Tübingen University Hospital at 7:00 a.m. the next morning, following a standardized overnight fast of approximately 12 hours. fMRI measurements were conducted on two separate days in a randomized, cross-over design (during the follicular phase and the luteal phase of the menstrual cycle; Fig. 1). Resting-state fMRI scans were acquired before and 30 min after INI on each day. Before and after INI, participants rated their hunger on a 0–10 visual analog scale (VAS; 0 = “not hungry at all,” 10 = “very hungry”) and completed the modified German version of the Food Craving Questionnaire-State (FCQ-S)^67^. Participants received 160U of intranasal insulin (Insulin Actrapid; Novo Nordisk, Bagsvaerd, Denmark). The spray was delivered over 4 minutes, with two puffs per nostril administered every minute. The visual food cue task was performed in the scanner 40 min after INI^14^.

The cycle phase was determined in a two-step approach that integrated calendar-based counting and serum sex hormone measurements^4^. Cycle day for each participant was determined retrospectively by accounting for individual cycle lengths and recording the onset of the current and subsequent menstrual cycles. The first day of menstrual bleeding was designated as day 1 of the follicular phase. The follicular phase was defined as beginning 2 days after menstruation onset and extending to a maximum of 17 days prior to the next cycle. The luteal phase was defined as occurring between 3 and 10 days before the onset of the subsequent cycle. Cycle phase assignments were further verified by measuring circulating levels of LH, FSH, estradiol, and progesterone. On each measurement day, blood samples were taken before the fMRI measurement to verify the respective cycle phase.

### MRI data acquisition

MRIs were conducted using a 3T Magnetom Prisma MRI scanner system (Siemens, MAGNETOM Prisma fit), with a 20-channel head coil. High-resolution anatomical images were collected using a T1-weighted MPRAGE sequence with a resolution of 1 × 1 x 1 mm^3^.

### Resting-state fMRI

Resting-state images were acquired using a T2*-weighted echo-planar-imaging (EPI) sequence with the following parameters: repetition time (TR) = 1180 ms, echo time (TE) = 34 ms, flip angle = 65°, bandwidth = 1848 Hz/pixel, echo spacing = 0.65 ms, voxel size = 2.5 × 2.5 × 2.5 mm^3^, 60 axial slices. Each functional run contained 250 image volumes. During the resting-state measurement, participants were instructed to keep their eyes closed and not to focus on anything in particular.

### Task-based fMRI

Images were obtained during the food cue task using another EPI sequence with the following parameters: TR = 1500 ms; TE = 34 ms; flip angle = 70°; bandwidth = 2264 Hz/pixel, echo spacing = 0.55 ms; voxel size 2 × 2 × 2 mm^3^; 72 axial slices. Each functional run contained 220 image volumes.

The visual food cue task included two sessions of 5 minutes and 30 seconds each with an event design^14^. Briefly, during the measurement, participants were presented with a set of 60 food images containing 15 high-caloric sweet (e.g., cakes), 15 high-caloric savory (e.g., pizza), 15 low-caloric sweet (e.g., fruits), and 15 low-caloric savory (e.g., vegetables) foods. These pictures were presented in a pseudo-randomized order. Each image was displayed for 2 seconds with an interstimulus interval of 6–10 seconds in a pseudorandomized order. Between the food images, a grey screen with a black fixation circle or, every 6–7 images, a black fixation cross, was shown at the center of the screen. To ensure participants’ attention, they were instructed to focus on the images and press a button immediately when a cross appeared between the pictures.

### fMRI data processing

#### Resting-state fMRI data

Preprocessing of resting-state data was performed in the CONN toolbox (https://www.nitrc.org/projects/conn). The steps in the pipeline were performed with default settings and included functional realignment and coregistration, slice-timing correction, outlier identification, segmentation and normalization, and functional smoothing^68^.

Group-level independent component analysis (GICA) was performed to extract spatially distinct functional resting-state networks. Preprocessed data were further analyzed using GIFT Toolbox v. 4.0b (https://trendscenter.org/trends/software/gift/). Data dimensionality was reduced to 36 temporal dimensions using Principal Component Analysis, followed by the infomax algorithm to estimate the independent components. Individual subject maps were then back-reconstructed and converted to z-scores for second-level analysis. These z-scores reflect the fit of a given voxel’s signal time course to the group-averaged component’s time course, revealing temporally correlated independent component networks. Further statistical analysis allows to compare functional connectivity changes within these networks between different conditions. Thirty-six independent components were identified by visual inspection of the spatial maps and power spectra, of which 16 were identified as noise artifacts. The components were then evaluated using spatial sorting, comparing them to the networks identified by Yeo et al.^69^ through spatial correlations in GIFT. Additionally, the anatomical regions represented in each spatial map were visually compared to previously described intrinsic connectivity networks^70–72^. As a result, 20 functional networks were identified (Supplementary Fig. S2).

#### Task-based fMRI data

Preprocessing of the task-based fMRI data was conducted using SPM12 (http://www.fil.ion.ucl.ac.uk/spm). The steps included slice timing, realignment, coregistration, normalization to MNI space, and Gaussian smoothing with a 6 mm FWHM. Participants with head movements exceeding 2° or 2 mm in any direction were excluded. High-pass filtering (128 s) and correction for global AR^1^ autocorrelation were also performed.

The separate brain response to high-caloric sweet, high-caloric savory, low-caloric sweet, and low-caloric savory food images were convolved with a canonical hemodynamic response function and then added into the general linear model, as recently described^14^. The six motion parameters were included in the model as confounders. The following contrasts were generated: sweet (30 images with high-caloric and low-caloric sweet foods combined) minus savory (30 images with high-caloric and low-caloric savory foods combined) food images; high-caloric (30 images with sweet and savory combined) minus low-caloric (30 images with sweet and savory combined) food images; all food images (combined 60 images). The individual contrasts were entered into a full factorial design for second-level analysis.

### Laboratory measurements

Participants were instructed to restrain from heavy physical activity, smoking, and alcohol within the 24 h before the experiments and fasted overnight prior to blood sample collection. HbA1c was quantified on the Tosoh glycohemoglobin analyzer (Tosoh Bioscience Tokyo, Japan) from fresh EDTA whole blood samples. Serum was allowed to clot in an upright position for 25 min at room temperature and then placed on ice prior to centrifugation (3100 *g* for 7 min) and hormone measurements. Insulin, C-peptide, estradiol, and progesterone measurements were performed using ADVIA Centaur XPT. All analytes were kept on ice and immediately measured in the routine diagnostic laboratory at the University Hospital Tübingen, accredited by the German Accreditation Body (DAkkS).

### Statistical analysis

#### Resting-state fMRI data

Second-level analysis was conducted in SPM12 to investigate changes in the 20 resting-state functional connectivity (FC) networks. The z-transformed spatial maps of each of the identified networks were included in a flexible factorial design with the factors including subject, cycle phase (follicular vs. luteal), and insulin administration (pre vs. post). An uncorrected threshold of *p* < 0.001 and a cluster-level family-wise error (FWEc) corrected threshold of *p* < 0.05 at a whole-brain level were applied. In addition, small volume corrections (SVC) were applied for the striatum (combined pallidum, putamen, and caudate), insula, hippocampus, dlLPFC and medial orbitofrontal cortex (mOFC) based on previous findings on brain insulin action using INI^6,73^. The masks were created using the Automated Anatomical Labeling (AAL) atlas via the WFU PickAtlas toolbox (https://www.nitrc.org/projects/wfu_pickatlas/). Bonferroni correction was applied for the number of regions of interest (ROIs), using a threshold of *p* < 0.01 (0.05/5).

FC values (beta-value) of significant clusters and voxels were extracted for further post-hoc analysis and effects of sex hormones. Two-sided paired t-tests were used to compare functional connectivity measures before (pre) and after (post) spray administration for each phase. These analyses were carried out using R version 4.3.1 (2023-06-16). Bonferroni correction was applied for the number of post hoc tests, with a threshold of *p* < 0.008 (0.05/6).

#### Exploratory analysis of sex hormones effects on resting-state functional connectivity networks

Linear mixed models were used to evaluate whether sex hormones (independent variable) are significant predictors for FC (dependent variable) across both cycle phases. For the interaction effect between sex hormones and INI, linear mixed models were used with an interaction term for INI (pre versus post spray) * sex hormone, entered as a fixed effect. Participants were modeled with a random intercept in every model. Log-transformed hormone values were used.

### Task-based fMRI data

#### Whole-brain analysis

Second-level analysis was performed in SPM12. The contrast of sweet minus savory food, high-caloric minus low-caloric food, and all food images minus implicit baseline were entered separately into a paired t-test model to examine the differences in brain food cue reactivity between the follicular and luteal phase at the whole-brain level. Results were thresholded at a voxel-level of *p* < 0.001 (uncorrected) and at a cluster-level of *p* < 0.05 (FWE-corrected for multiple comparisons).

#### Exploratory ROI analysis

In an ROI analysis, we focused on brain areas that have previously been identified as insulin sensitive as well as responsive to food cues, including the bilateral hippocampus, dorsal striatum, ventral striatum, amygdala, insula, dlPFC, mOFC^6,73^. The BOLD signal was extracted from these regions based on the contrasts described above. Two-sided, paired t-tests were applied to test for differences in food cue activity between the follicular and the luteal phase using R version 4.3.1. The masks were created using the AAL atlas via the WFU PickAtlas toolbox (https://www.nitrc.org/projects/wfu_pickatlas/).

#### Exploratory analysis of sex-hormone effects

Associations between sex hormone levels (log-transformed) and neural food cue response (beta value) were analyzed using linear mixed models. The brain activity measure was modeled as the dependent variable and hormone levels as the independent variable, with participants included as random intercepts.

### Effect of cycle phase on food craving

Linear mixed models were used to examine the effects of menstrual cycle phases (follicular vs. luteal) on hunger ratings and food craving, including subscales of desire, hunger, control, relief, and reinforcement, as well as the total craving score. The interaction between cycle phase and INI (pre vs. post spray) was also assessed.

### Reporting summary

Further information on research design is available in the Nature Portfolio Reporting Summary linked to this article.

## Supplementary information

Supplementary Information

Description of Additional Supplementary Files

Supplementary Data 1

Reporting Summary

## Data Availability

Numerical source data for graphs can be obtained in Supplementary Data 1. Raw MRI data can be obtained on the OpenNeuro platform (10.18112/openneuro.ds006893.v1.0.3). All other data are available from the corresponding author on reasonable request.
