# Supplementary material for: The interplay of central insulin and menstrual cycle on functional brain networks and neural food cue reactivity in women
Source: Commun Biol. 2026 Jan 6;9:76. doi: 10.1038/s42003-025-09341-9 (PMC12820398; doi:10.1038/s42003-025-09341-9)
Supplement: Supplementary file 1 — Supplementary Information [file 42003_2025_9341_MOESM1_ESM.pdf]

Supplementary Figure 1

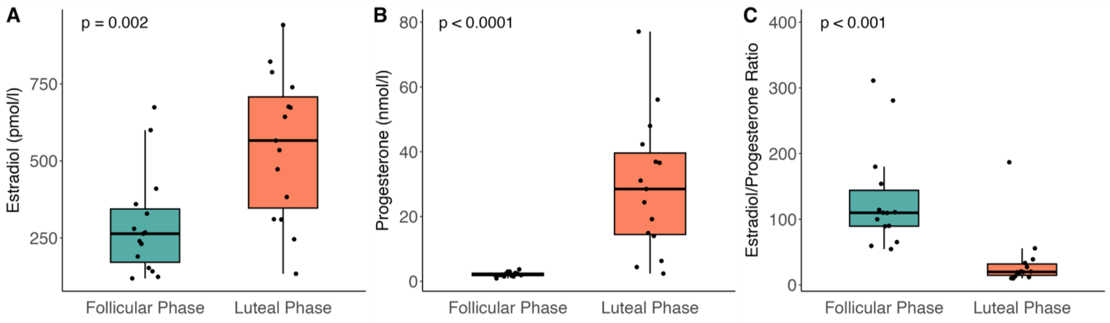

Supplementary Figure 1: Differences in sex hormone levels between the phases of the menstrual cycle

Serum concentrations of ***(A)*** estradiol and ***(B)*** progesterone were higher in the luteal phase, while the ***(C)*** estradiol/progesterone ratio was higher in the follicular phase of the menstrual cycle. Presented are tukey box plots with whiskers extending to 1.5 times the interquartile range and individual data points. p-values are derived from two-sided paired t-tests for the comparison of log transformed sex hormon e levels between the phases.

Supplementary Figure 2

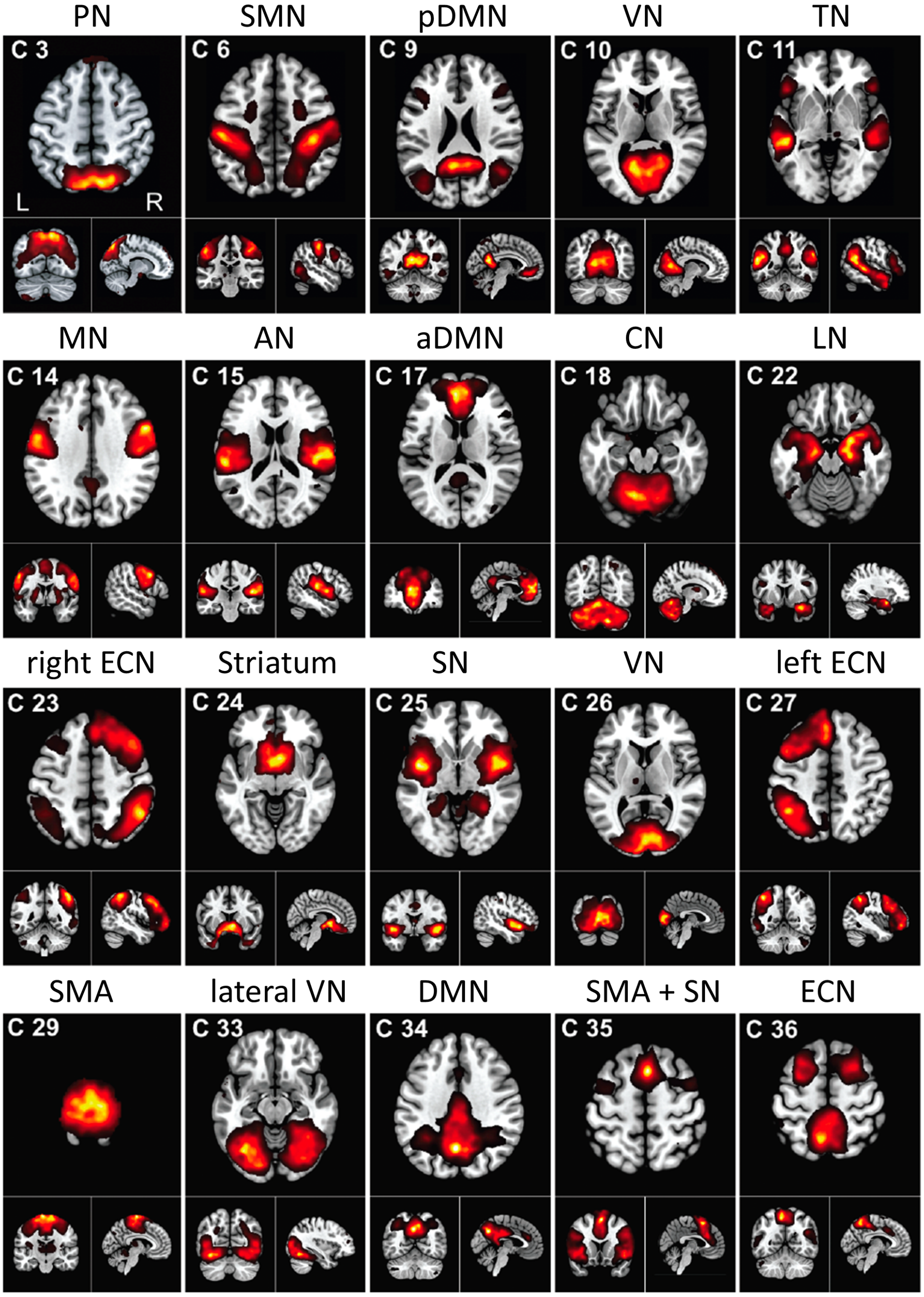

Supplementary Figure 2: Group-averaged resting state networks extracted from fMRI data of all subjects thresholded at $p < 0.05$ (FWE-corrected) are shown . Orthogonal slices of the peak cluster values are presented in standard (MNI152) brain space. Abbreviations: aDMN = Anterior Default Mode Network; AN = Auditory Network; CN = Cerebellar Network; DMN = Default Mode Network; ECN = Executive Control Network; LN = Limbic Network; MN = Motor Network; pDMN = Posterior Default Mode Network; PN = Parietal Network; SMA = Su pplementary Motor Area; SMN = Sensorimotor Network; SN = Salience Netw ork; Striatum = Striatal Network; TN = Temporal Network; VN = Visual Network. All analyses included n = 15 participants.

Supplementary Figure 3

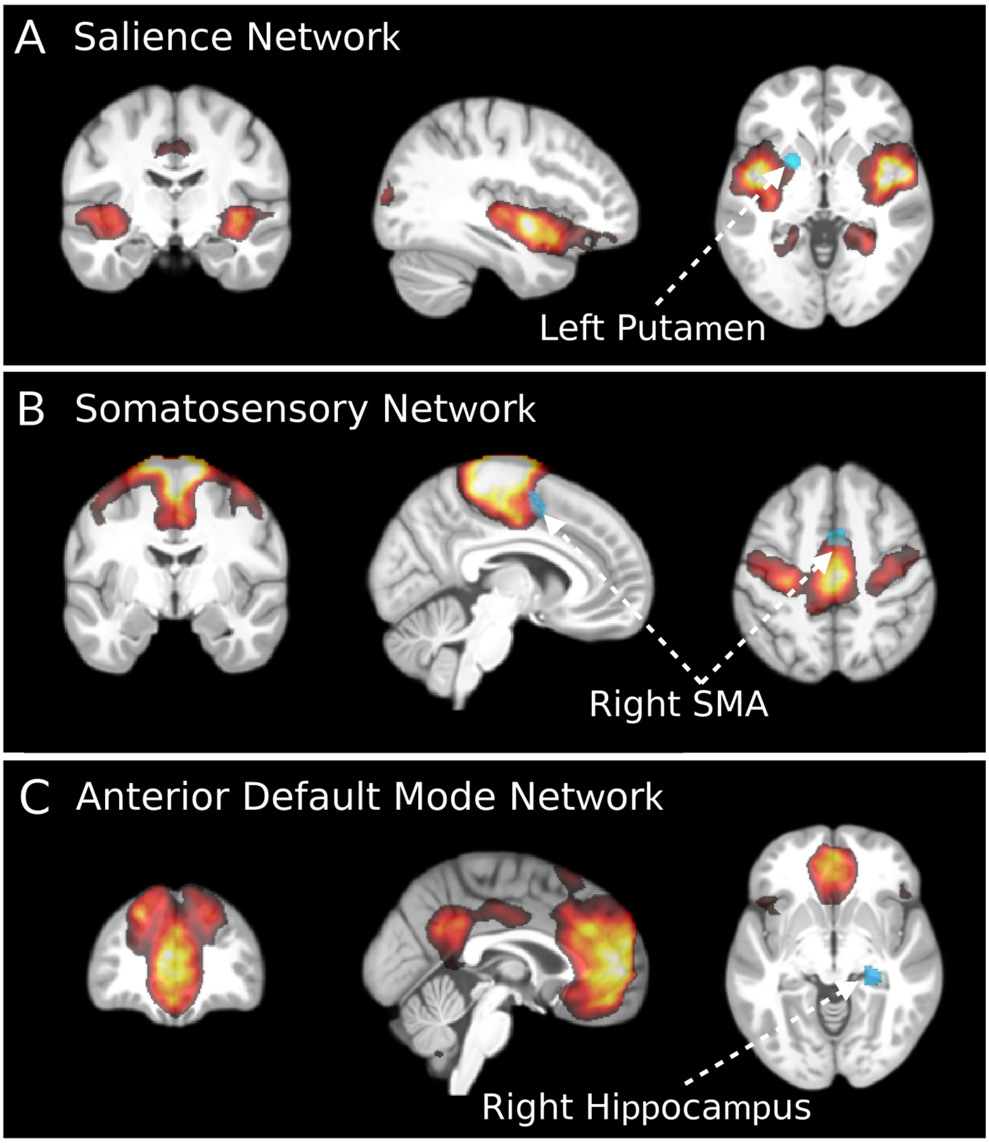

Supplementary Figure 3: Interaction effect of insulin administration and menstrual cycle phase in the ***(A)*** putamen of the salience network (pFWE = 0.001, whole-brain corrected), ***(B)*** supplementary motor area (SMA) of the somatosensory network (pFWE = 0.014, whole-brain corrected) and ***(C)*** hippocampus of the anterior default mode network (pFWE = 0.007, small volume correction). The color map corresponds to network masks and t values ($p < 0.001$ uncorrected) overlaid on the standardized T1 image. All analyses included n = 15 participants.
