# Supplementary material for: The interplay of central insulin and menstrual cycle on functional brain networks and neural food cue reactivity in women
Source: Commun Biol. 2026 Jan 6;9:76. doi: 10.1038/s42003-025-09341-9 (PMC12820398; doi:10.1038/s42003-025-09341-9)
Supplement: Supplementary file 4 — Reporting Summary [file 42003_2025_9341_MOESM4_ESM.pdf]

nature portfolio

Corresponding author(s): Stephanie Kullmann

Last updated by author(s): Nov 5, 2025

Reporting Summary

Nature Portfolio wishes to improve the reproducibility of the work that we publish. This form provides structure for consistency and transparency in reporting. For further information on Nature Portfolio policies, see our Editorial Policies and the Editorial Policy Checklist.

Statistics

For all statistical analyses, confirm that the following items are present in the figure legend, table legend, main text, or Methods section.

n/a	Confirmed	
☐	☒	The exact sample size (*n*) for each experimental group/condition, given as a discrete number and unit of measurement
☐	☒	A statement on whether measurements were taken from distinct samples or whether the same sample was measured repeatedly
☐	☒	The statistical test(s) used AND whether they are one- or two-sided<br>*Only common tests should be described solely by name; describe more complex techniques in the Methods section.*
☐	☒	A description of all covariates tested
☐	☒	A description of any assumptions or corrections, such as tests of normality and adjustment for multiple comparisons
☐	☒	A full description of the statistical parameters including central tendency (e.g. means) or other basic estimates (e.g. regression coefficient) AND variation (e.g. standard deviation) or associated estimates of uncertainty (e.g. confidence intervals)
☐	☒	For null hypothesis testing, the test statistic (e.g. *F*, *t*, *r*) with confidence intervals, effect sizes, degrees of freedom and *P* value noted<br>*Give P values as exact values whenever suitable.*
☒	☐	For Bayesian analysis, information on the choice of priors and Markov chain Monte Carlo settings
☒	☐	For hierarchical and complex designs, identification of the appropriate level for tests and full reporting of outcomes
☒	☐	Estimates of effect sizes (e.g. Cohen's *d*, Pearson's *r*), indicating how they were calculated

Our web collection on statistics for biologists contains articles on many of the points above.

Software and code

Policy information about availability of computer code

Data collection	none
Data analysis	R (version 4.3.1). R packages: G*power (version 3.1). SPM12 (Wellcome Trust Centre for Neuroimaging); CONN toolbox (https://www.nitrc.org/projects/conn; GIFT Toolbox v. 4.0b (https://trendscenter.org/trends/software/gift/).

For manuscripts utilizing custom algorithms or software that are central to the research but not yet described in published literature, software must be made available to editors and reviewers. We strongly encourage code deposition in a community repository (e.g. GitHub). See the Nature Portfolio guidelines for submitting code & software for further information.

Data

Policy information about availability of data

All manuscripts must include a data availability statement. This statement should provide the following information, where applicable:

- Accession codes, unique identifiers, or web links for publicly available datasets
- A description of any restrictions on data availability
- For clinical datasets or third party data, please ensure that the statement adheres to our policy

The data generated during the current study are shared with researches upon reasonable request. Requests will be promptly reviewed by the Data Access Steering Committee of the Institute for Diabetes Research and Metabolic Diseases, Tübingen, Germany. Any data and materials that can be shared will be released via a Material Transfer Agreement.

Research involving human participants, their data, or biological material

Policy information about studies with human participants or human data. See also policy information about sex, gender (identity/presentation), and sexual orientation and race, ethnicity and racism.

Reporting on sex and gender	In our previous work, we found sex specific differences of brain insulin action and how it relates to behavior and peripheral metabolism (Zhao et al. JCEM 2024; Hummel et al. Nature Metab 2023; Wagner et al. Diab Obes Metab 2023). First findings show that the menstrual cycle plays a prominant role how the brain regulates metabolism (Hummel et al. Nature Metab 2023), We now studied the interaction of menstrual cycle and brain insulin action on functional brain networks and neural food cue response to study the underlying neural pathway. The biological sex of the participants was ensured by the sex hormone analysis performed to determine the menstrual cycle phase.
Reporting on race, ethnicity, or other socially relevant groupings	Participants with high levels of education and a high socio-economic status are enriched in this study population. Given the repeated measurement design, we do not expect a strong impact on the results.
Population characteristics	We studied young, lean, healthy, naturally cycling women.
Recruitment	Participants were recruited by University-wide email anouncements. Therefore, participants with high levels of education and a high socio-economic status are enriched in this study population. This could be a selection bias. The repeated measurement design minimize the impact of this bias.
Ethics oversight	The study (NCT03929419) received approval by the local ethics committee (Ethics Committee of the Medical Faculty of the Eberhard Karls University and the University Hospital Tübingen) and was conducted according to the relevant guidelines and regulations.

Note that full information on the approval of the study protocol must also be provided in the manuscript.

Field-specific reporting

Please select the one below that is the best fit for your research. If you are not sure, read the appropriate sections before making your selection.

☒ Life sciences ☐ Behavioural & social sciences ☐ Ecological, evolutionary & environmental sciences

For a reference copy of the document with all sections, see nature.com/documents/nr-reporting-summary-flat.pdf

Life sciences study design

All studies must disclose on these points even when the disclosure is negative.

Sample size	Sample size was selected to provide sufficient power (1-β = .86) for moderate within-group effects (dz = .40) on the basis of previous investigations of brain insulin action.
Data exclusions	One participants was excluded as she did not allocate to the correct cycle phase. For the task-based fMRI analysis, three participants were further excluded due to incomplete fMRI measurements or insufficient data quality. One ratio of estradiol/progesterone measurement was identified as an outlier (> 3 SD above the mean) and was therefore excluded from sex hormone analysis for both resting-state and task-based data.
Replication	We confirm that the regions identified to be responsive to intranasal insulin in the current study we found in previous fMRI studies (by our and other groups) Ref: Kullmann et al Lancet D&E 2020; Nijssen et al 2023 Neuroendocrinology)
Randomization	The initial cycle phase for the experiments was chosen in a random order.
Blinding	Persons performing fMRI data acqusition and fMRI data analysis were blinded to cycle phase .

Reporting for specific materials, systems and methods

We require information from authors about some types of materials, experimental systems and methods used in many studies. Here, indicate whether each material, system or method listed is relevant to your study. If you are not sure if a list item applies to your research, read the appropriate section before selecting a response.

Materials & experimental systems

n/a	Involved in the study
☒	☐ Antibodies
☒	☐ Eukaryotic cell lines
☒	☐ Palaeontology and archaeology
☒	☐ Animals and other organisms
☐	☒ Clinical data
☒	☐ Dual use research of concern
☒	☐ Plants

Methods

n/a	Involved in the study
☒	☐ ChIP-seq
☒	☐ Flow cytometry
☐	☒ MRI-based neuroimaging

Clinical data

Policy information about clinical studies

All manuscripts should comply with the ICMJE guidelines for publication of clinical research and a completed CONSORT checklist must be included with all submissions.

Clinical trial registration: NCT03929419

Study protocol: The study protocol is available as a supplemental file in the publication Hummel et al, 2023 Nature Metabolism

Data collection: Data were collected at the University Hospital of Tübingen between April 2019 and March 2021.

Outcomes: The primary outcome of the study was published in Hummel et al. 2023, Nature Metabolism.
In this study secondary outcomes are evaluated:
1. Effect of central insulin on functional brain networks in the follicular and the luteal phase of the mentstrual cycle.
Resting state fMRI measurements were performed before and after intranasal insulin administration on two measurement days, i.e. in the follicular and the luteal phase. Hence, we can evaluate the interaction between menstriual cycle and brain insulin action.
2. Neural food cue response in the follicular and luteal phase of the menstrual cycle. BOLD signal was measured in response to visual food cues.

Plants

Seed stocks: *Report on the source of all seed stocks or other plant material used. If applicable, state the seed stock centre and catalogue number. If plant specimens were collected from the field, describe the collection location, date and sampling procedures.*

Novel plant genotypes: *Describe the methods by which all novel plant genotypes were produced. This includes those generated by transgenic approaches, gene editing, chemical/radiation-based mutagenesis and hybridization. For transgenic lines, describe the transformation method, the number of independent lines analyzed and the generation upon which experiments were performed. For gene-edited lines, describe the editor used, the endogenous sequence targeted for editing, the targeting guide RNA sequence (if applicable) and how the editor was applied.*

Authentication: *Describe any authentication procedures for each seed stock used or novel genotype generated. Describe any experiments used to assess the effect of a mutation and, where applicable, how potential secondary effects (e.g. second site T-DNA insertions, mosiacism, off-target gene editing) were examined.*

Magnetic resonance imaging

Experimental design

Design type: resting-state fMRI and event related task based fMRI

Design specifications: Participants were presented a set of 60 food images containing 15 high-caloric sweet (e.g., cakes), 15 high-caloric savory (e.g., pizza), 15 low-caloric sweet (e.g., fruits), and 15 low-caloric savory (e.g., vegetables) foods. These pictures were presented in a pseudo-randomized order. Each image was displayed for 2 seconds with an interstimulus interval of 6–10 seconds in a pseudorandomized order. Between the food images, a grey screen with a black fixation circle or, every 6–7 images, a black fixation cross, was shown at the center of the screen.

Behavioral performance measures: To ensure participants' attention, they were instructed to focus on the images and press a button immediately when a cross appeared between the pictures.

Acquisition

Imaging type(s): functional

Field strength: 3 Tesla

Sequence & imaging parameters: Resting-state images were acquired using a T2*-weighted echo-planar-imaging (EPI) sequence with the following

Sequence & imaging parameters

parameters: repetition time (TR) = 1180 ms, echo time (TE) = 34 ms, flip angle = 65°, bandwidth = 1848 Hz/pixel, echo spacing = 0.65 ms, voxel size = 2.5 × 2.5 × 2.5mm3, 60 axial slices. Each functional run contained 250 image volumes. During the food cue task, images were obtained using another EPI sequence with the following parameters: TR = 1500 ms; TE = 34 ms; flip angle = 70°; bandwidth = 2264 Hz/pixel, echo spacing = 0.55 ms; voxel size 2 × 2 × 2 mm3; 72 axial slices. Each functional run contained 220 image volumes.

Area of acquisition

Whole Brain

Diffusion MRI ☐ Used ☒ Not used

Preprocessing

Preprocessing software

Preprocessing of resting-state data was performed in the CONN toolbox (https://www.nitrc.org/projects/conn). The steps in the pipeline were performed with default settings and included functional realignment and coregistration, slice-timing correction, outlier identification, segmentation and normalization, and functional smoothing.
Preprocessing of the task-based fMRI data was conducted using SPM12 (http://www.fil.ion.ucl.ac.uk/spm). The steps included slice timing, realignment, coregistration, normalization to MNI space and Gaussian smoothing with a 6mm FWHM. High-pass filtering (128 s) and correction for global AR (1) autocorrelation were also performed.

Normalization

The high resolution T1-weighted image was normalized in Montreal Neurological Institute space (1 x 1 x 1 mm) using SPM12's unified segmentation normalization, and the resulting parameter file was used with the individual coregistered functional image in normalized space (3 x 3 x 3 mm).

Normalization template

MNI305

Noise and artifact removal

Participants with head movements exceeding 2° or 2mm in any direction were excluded. For resting-state fMRI, thirty-six independent components were identified by visual inspection of the spatial maps and power spectra of which 16 were identified as noise artifacts.

Volume censoring

NA

Statistical modeling & inference

Model type and settings

For resting-state fMRI, preprocessed data were further analyzed using GIFT Toolbox v. 4.0b (https://trendscenter.org/trends/software/gift/).

Effect(s) tested

For resting-state fMRI: flexible factorial design with the factors including subject, cycle phase (follicular vs. luteal), and insulin administration (pre vs. post) was used. For task based fMRI, the contrast of sweet minus savory food, high-caloric minus low-caloric food and all food images minus implicit baseline were entered separately into a paired t-test model to examine the differences in brain food cue reactivity between the follicular and luteal phase at whole-brain level.

Specify type of analysis: ☐ Whole brain ☐ ROI-based ☒ Both

Anatomical location(s)

In a ROI analysis we focused on brain areas which have previously been identified as insulin sensitive as well as responsive to food cues, including the bilateral hippocampus, dorsal striatum, ventral striatum, amygdala, insula, dlPFC, mOFC The masks were created based on the wfu pick atlas (https://www.nitrc.org/projects/wfu_pickatlas/).

Statistic type for inference

(See Eklund et al. 2016)

cluster-wise correction for whole brain analyses

Correction

FWE

Models & analysis

n/a	Involved in the study
☒	☐ Functional and/or effective connectivity
☒	☐ Graph analysis
☐	☒ Multivariate modeling or predictive analysis

Multivariate modeling and predictive analysis

Group-level independent component analysis (GICA) was performed to extract spatially distinct functional resting-state networks. Data dimensionality was reduced to 36 temporal dimensions using Principal Component Analysis followed by the infomax algorithm to estimate the independent components. Individual subject maps were then back-reconstructed and converted to z-scores for second-level analysis, revealing temporally correlated independent component networks. 36 independent components were identified by visual inspection of the spatial maps and power spectra of which 16 were identified as noise artifacts. The components were then evaluated using spatial sorting, comparing them to the networks identified by Yeo et al. through spatial correlations in GIFT. Additionally, the anatomical regions represented in each spatial map were visually compared to previously described intrinsic connectivity networks. As a result, 20 functional networks were identified.
